# Challenges and surprises that arise with nucleic acids during model building and refinement

**DOI:** 10.1107/S0907444912001084

**Published:** 2012-03-16

**Authors:** William G. Scott

**Affiliations:** a Department of Chemistry and Biochemistry and the Center for the Molecular Biology of RNA, University of California at Santa Cruz, Santa Cruz, CA 95064, USA

**Keywords:** nucleic acids, model building, refinement

## Abstract

The process of building and refining crystal structures of nucleic acids, although similar to that for proteins, has some peculiarities that give rise to both various complications and various benefits. Although conventional isomorphous replacement phasing techniques are typically used to generate an experimental electron-density map for the purposes of determining novel nucleic acid structures, it is also possible to couple the phasing and model-building steps to permit the solution of complex and novel RNA three-dimensional structures without the need for conventional heavy-atom phasing approaches.

## Abstract 2

The challenges that arise in nucleic acid model building as a consequence of their simpler and more symmetric super-secondary structures are addressed.

## 1. Introduction

Although it would be an over-exaggeration (to invoke Edward Abbey’s delightful turn of phrase) to suggest that model building and refinement of nucleic acid crystal structures differs fundamentally from that of protein structures, there are some peculiarities, as well as both advantages and disadvantages, that are worthy of consideration should you happen to find yourself solving the crystal structure of a nucleic acid, either by itself or bound to a protein ligand. Macromolecular crystallographic diffraction behaves almost identically, and crystal structures are experimentally phased by the same MIR/MAD approaches, whether they contain protein, nucleic acid or both. Refinement and validation typically proceed in a similar manner, and all of the commonly used refinement and model-building software handles both types of polymers with ease. Most of the challenges that arise in nucleic acid model building are a consequence of their simpler and more symmetric super-secondary structures (*i.e.* double helices and variations). Most DNA and DNA–protein crystal structures involve quite regular Watson–Crick base-paired DNAs. RNA structures in general are more complicated, but even highly globular RNA structures, such as those of the ribosome, are comprised largely of regions of A-form or distorted A-form helices embedded in a more complex fold. As a consequence, all nucleic secondary-structural elements tend to appear quite similar, making sequence assignment and backbone tracing somewhat daunting. The need for accurate sequence data and biochemical constraints to augment and double-check a crystallographically derived structure is thus quite significant. We have recently found that paradoxically this same structural ambiguity can actually aid in solving complex RNA structures.

## 2. Nucleic acid super-secondary structures

All organisms, apart from RNA viruses and single-stranded DNA viruses, possess a genome comprised exclusively of Watson–Crick base-paired double-stranded DNA that possesses a highly regular super-secondary structure: B-form (or occasionally A-form) nucleic acid. Although the sequence is irregular, the Watson–Crick base pairs, as is well known, are isosteric and the sugar-phosphate backbone is completely regular. Hence, apart from bending and other (typically localized) helical irreg­ularities, all double-stranded DNAs adhere to essentially identical B-­form, or occasionally A-form, helical structures.

Although the structures of RNAs tend to be less regular, containing various loops, bulges, noncanonical base pairs and tertiary contacts, RNAs in general also adhere rather closely to overall A-form super-secondary structures. Unlike DNAs, these A-­form helices typically fold back on themselves, creating complex tertiary structures as observed in tRNA, in many of the larger ribozymes and, most extensively, in ribosomal RNA. Nevertheless, even ribosomal RNAs are dominated by Watson–Crick base-paired secondary-structural elements and are thus to a reasonable approximation merely clusters of A-form nucleic acid super-secondary-structural elements (Noller & Woese, 1981 ▶).

## 3. Nucleic acid tertiary structures

RNA, unlike most DNA, may possess a complex tertiary structure. Comparatively small RNAs, such as the approximately 75-nucleotide tRNA, are rather globular and larger structural RNAs, such as ribozymes and the ribosome, are in many ways more reminiscent of proteins than nucleic acids. It is the tertiary-structural richness enabled by 2′-OH-mediated contacts and tertiary base pairs that permits ribozymes and the ribosome to possess catalytic activity that rivals (and in the case of the ribosome surpasses) that of globular protein enzymes.

Yet, a close examination of the known complex RNA tertiary structures reveals a simplicity that is absent in most protein structures. Because most structured RNA forms significant regions of either perfect or at least near-perfect A-­form helical elements, the tertiary structures of RNAs tend to be little more than large assemblies of A-form helical elements. Proteins tend to have super-secondary-structural elements comprised of regions of β-sheets or α-helices, and individual domains or subunits consist of assemblies of these super-secondary-structural elements, as well as a fair amount of more irregular structural regions such as connecting loops. RNA structures, in this sense, are less complex.

## 4. tRNA: a classic example

The first crystal structure of a nucleic acid appeared in 1974 in the form of yeast phenylalanine tRNA at 3 Å resolution. Two rival research groups focused on an orthorhombic and a closely related monoclinic form of the same molecule. The group working on the orthorhombic form published an erroneous structure (Suddath *et al.*, 1974 ▶) and corrected it in a subsequent publication (Kim *et al.*, 1974 ▶) at the same time that the correct monoclinic structure (Robertus *et al.*, 1974 ▶) was first published.

tRNAs all possess a rather simple cloverleaf-like secondary structure consisting of four helical stems, three of which are capped by loops (including the anticodon loop). The four helical stems in fact form two quasi-continuous double-helical super-secondary structures that then fold and pack at a roughly 90° angle, yielding a remarkably complex tertiary structure for an RNA comprised of only 74 nucleotides. It is noteworthy that in the tRNA structure only three of the 74 nucleotides are not involved in helical stacking interactions.

**Figure 1:**
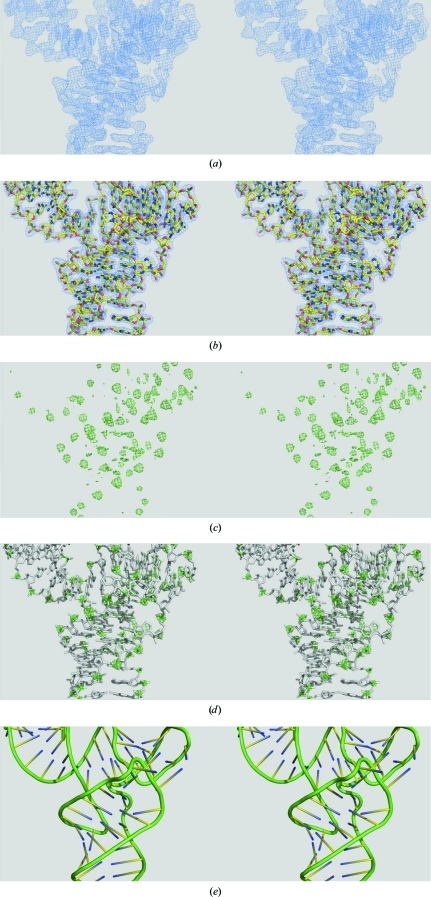
Wall-eyed stereoviews of yeast phenylalanine tRNA. (*a*) A σ_A_-weighted *F*_o_ − *F*_c_ electron-density map contoured at 1.0 r.m.s. at 2 Å resolution shown as a blue mesh. (*b*) The same map with the atomic model imposed on the density. (*c*) The same map but contoured at 5.0 r.m.s., revealing electron-rich regions that typically correspond to phosphate density, shown as a green mesh. (*d*) The same map as (*c*) with the atomic model imposed. (*e*) A cartoon ribbon diagram showing the correct phosphodiester backbone trace in green.

A significant portion of the monoclinic tRNA paper (Robertus *et al.*, 1974 ▶) is devoted to pointing out how errors in tracing the backbone of the tRNA in the orthorhombic structure went astray and how the absence of the phylogenetically predicted tertiary base pair and triples (Levitt, 1969 ▶; Klug *et al.*, 1974 ▶) was a key indicator that the first ortho­rhombic tRNA crystal structure was in error. This is a particularly instructive as well as a pertinent insight. RNA electron density, even very high-quality modern 2 Å resolution density (obtained from the same monoclinic tRNA crystals; Jovine *et al.*, 2000 ▶) can be quite disorienting, as illustrated in Fig. 1 ▶. A refined 2*F*_o_ − *F*_c_ σ_A_-weighted electron-density map for the central portion of the tRNA molecule contoured at 1.0 r.m.s. is shown in Fig. 1 ▶(*a*) and the refined atomic model of the tRNA is imposed upon the density in Fig. 1 ▶(*b*). Without the aid of the molecular structure, it is clear that the 2 Å refined 2*F*_o_ − *F*_c_ σ_A_-weighted map is potentially more confusing and ambiguous than that of a typical globular protein at 2 Å resolution. The potential for making a ‘wrong turn’ in the trace of the phosphodiester backbone is readily apparent.

However, the electron-density map does possess some characteristics that make it a bit easier to interpret than that of a protein. Each nucleotide contains an electron-rich phosphate group (a P atom and four O atoms). Well ordered phosphates are thus much more electron-dense than the rest of the nucleic acid. The same 2*F*_o_ − *F*_c_ map contoured at 5.0 r.m.s. is depicted in Fig. 1 ▶(*c*). Most of the green peaks correspond to phosphates, permitting a reasonably objective double-check of the backbone trace and assignment. The all-atom structure is imposed upon the density in Fig. 1 ▶(*d*) and Fig. 1 ▶(*e*) shows an abstraction of the backbone as a green tube.

The original tRNA crystal structures thus reveal the need for great care in the initial model-building phase, as well as the need to account for all of the biologically relevant data (such as the requirement to explain the Levitt phylogenetically derived invariant tertiary base pair and triples and the requirement to maintain an approximately A-form helical super-secondary structure).

## 5. RNA and the crystallographic phase problem

The macromolecular crystallographic phase problem and its solution (Muirhead & Perutz, 1963 ▶) are essentially the same for protein and nucleic acid crystal structures; the physics of diffraction is identical in both cases and heavy-atom isomorphous replacement methods are required to phase novel crystal structures in both cases. Nucleic acids have fewer unique reactive functional groups than proteins, so in practice obtaining good derivatives is often more challenging. However, recent progress creating binding sites for Ir(NH_3_)_6_^3+^ and Os(NH_3_)_6_^3+^(Keel *et al.*, 2007 ▶), as well as synthetic incorporation of modified nucleotides such as 5-bromouracil (5BrU) or selenium-substituted nucleotides (Serganov *et al.*, 2005 ▶), have made the heavy-atom isomorphous replacement approach to phasing nucleic acids much more tractable. In addition, Se, Ir, Os and Br all have useful X-ray absorption edges, increasing their utility for phasing based upon anomalous dispersion and absorption. Engineered heavy-atom binding sites also significantly simplify the task of assigning the nucleic acid sequence to the electron density, since they are incorporated at known positions in the sequence. Although the phosphorus anamolous signal has been proposed for phasing, in practice it seems to be too weak to make a useful contribution. However, it can have some utility when attempting to differentiate a phosphate peak from other strong features in an experimental map when one is trying to trace the backbone of a nucleic acid.

## 6. Model building: do we really need experimental phases?

Conventional wisdom holds that molecular-replacement methods are not effective for solving the phase problem of macromolecules with novel unique tertiary structures. Even NMR structures are often found to be insufficiently similar for use as a probe for solving a crystal structure by molecular replacement (Chen *et al.*, 2000 ▶; Qian *et al.*, 2007 ▶). However, in the course of phasing a ligase ribozyme, we found that molecular replacement using a subset of idealized model A-form RNA helical fragments based on the known sequence, with no prior knowledge of their disposition in three-dimensional space, was sufficient for solving the phase problem for the crystal structure of this ribozyme (Robertson & Scott, 2007 ▶). The asymmetric unit was about the size of two tRNA molecules and it possessed no noncrystallographic symmetry (as the two molecules were found to be in radically different conformations). Our result indicates that, at least in principle, it is possible to solve novel nucleic acid structures without experimentally derived phases. Rather, the phases are in essence bootstrapped as an integral component of model building and refinement.

## 7. A general approach to solving novel RNA structures without heavy-atom derivatives

A known secondary structure consisting of Watson–Crick base-paired helices is usually available before one embarks upon the crystallographic structural determination of an RNA such as a tRNA, ribozyme or the ribosome. If not, programs such as *mFold* or *ViennaRNA* can give a reasonable estimate. The molecular-graphics display, modeling and refinement program *Coot* (*Crystallographic Object-Oriented Toolkit*; Emsley *et al.*, 2010 ▶) provides a very straightforward way to generate idealized model A-form RNA fragments using the menu item ‘calculate > other modelling tools > ideal DNA/RNA’ and the secondary-structural sequence of one strand of an RNA duplex. Doing so generates an ideal A-­form RNA helix for any given sequence. We have found that starting with up to four independent helical elements in four separately named PDB files gives the best results when employing the automated molecular-replacement program *Phaser* (McCoy *et al.*, 2005 ▶).

This is true even if the RNA represented by these fragments comprises less than half of the total RNA in the crystallo­graphic asymmetric unit. The resulting ‘under-sampling’ often improves the molecular-replacement solution. *Phaser* automatically attempts to arrange the RNA fragments in three-dimensional space in a way that yields the best molecular-replacement solution (and therefore the best phase estimate). Four ‘ENSEmble’ entries are required for the four substructure PDB files, four ‘COMPosition NUCLeic’ entries are required to designate these as nucleic acids and to assign them molecular weights (based upon their sequences) and four ‘SEARch ENSEmble’ entries are required to designate each as an independent simultaneous search model.

If this initial step is at all successful, the *Phaser*-calculated σ_A_-weighted 2*F*_o_ − *F*_c_ map will show weak or broken-up density where the model is incorrect and more convincing continuous density where the model is approximately correct. Typically, about one third to one half of the model will occupy reasonably strong density and about one third of the model will occupy weak or non-existent density. This initial model should be edited within *Coot*, mercilessly deleting any part of the model involved in a steric clash or that does not occupy reasonably convincing electron density. When this editing process is complete, there should be few if any atoms that do not occupy electron density and no steric clashes should remain. However, it is most likely that there is no plausible physical connectivity between subsets of the RNA sequence. This is because the molecular-replacement procedure we are using cannot resolve sequence details. Fortunately, it is in fact not necessary for the starting model to possess the correct sequence; all that is required is that each structural element represents an approximately correct secondary structure.

The edited molecular-replacement solution is then refined, typically using *REFMAC* (Murshudov *et al.*, 2011 ▶) within *Coot*, and used as a partial model for subsequent iterations of molecular replacement within *Phaser*. At this point simply including one additional helical element is usually sufficient for further model improvement; each addition requires further manual editing as described in the previous paragraph. When further addition of helical elements yields no further improvement in the electron-density map, the initial structure is refined using *REFMAC* and the resulting phase probability distributions need to be converted to Hendrickson–Lattmann coefficients using the *CCP*4 (Winn *et al.*, 2011 ▶) program *HLTOFOM*. These phases, when combined with the experimentally measured amplitudes, may then be treated as if they were determined by isomorphous replacement, with accompanying phase-error estimates. Specifically, improvement of the phases using solvent flattening will simultaneously reduce model bias and improve the electron-density map. The initial model used to generate the phases at this point is discarded. The newly solvent-flattened electron-density map may now be treated as if it is an initial experimental map.

**Figure 2:**
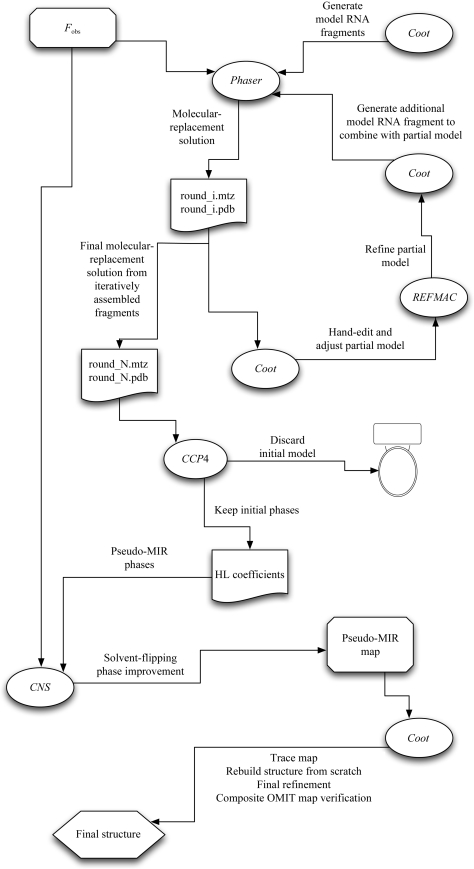
Schematic flowchart representation of the phasing procedure.

A flowchart that depicts the workflow described is shown in Fig. 2 ▶. Further details describing this procedure have been published elsewhere (Robertson & Scott, 2008 ▶; Robertson *et al.*, 2010 ▶).

## 8. Future prospects

Although the model-building/phasing/refinement approach to solving crystal structures has proven to be successful with RNAs and RNA–protein complexes (both unsolved and previously solved PDB depositions including all of the small self-cleaving ribozymes, several regulatory RNA elements and the U1A protein–RNA complex), there is no reason in principle why it might not be more widely applicable. In the case of protein structures, we now have a fairly complete structural library of protein folds and super-secondary structures, as well as cofactor structures. Thus, a similar approach that uses protein domains and cofactors as ‘atoms’ rather than A-form helices may also have potential.

## References

[bb1] Chen, Y. W., Dodson, E. J. & Kleywegt, G. J. (2000). *Structure*, **8**, R213–R220. 10.1016/s0969-2126(00)00524-4 11080645

[bb3] Emsley, P., Lohkamp, B., Scott, W. G. & Cowtan, K. (2010). *Acta Cryst.* D**66**, 486–501. 10.1107/S0907444910007493 PMC2852313 20383002

[bb4] Jovine, L., Djordjevic, S. & Rhodes, D. (2000). *J. Mol. Biol.* **301**, 401–414. 10.1006/jmbi.2000.3950 10926517

[bb5] Keel, A. Y., Rambo, R. P., Batey, R. T. & Kieft, J. S. (2007). *Structure*, **15**, 761–772. 10.1016/j.str.2007.06.003 PMC1995091 17637337

[bb6] Kim, S.-H., Suddath, F. L., Quigley, G. J., McPherson, A., Sussman, J. L., Wang, A. H.-J., Seeman, N. C. & Rich, A. (1974). *Science*, **185**, 435–440. 10.1126/science.185.4149.435 4601792

[bb7] Klug, A., Ladner, J. & Robertus, J. D. (1974). *J. Mol. Biol.* **89**, 511–516. 10.1016/0022-2836(74)90480-x 4613865

[bb8] Levitt, M. (1969). *Nature (London)*, **224**, 759–763. 10.1038/224759a0 5361649

[bb9] McCoy, A. J., Grosse-Kunstleve, R. W., Storoni, L. C. & Read, R. J. (2005). *Acta Cryst.* D**61**, 458–464. 10.1107/S0907444905001617 15805601

[bb10] Muirhead, H. & Perutz, M. F. (1963). *Nature (London)*, **199**, 633–638. 10.1038/199633a0 14074546

[bb11] Murshudov, G. N., Skubák, P., Lebedev, A. A., Pannu, N. S., Steiner, R. A., Nicholls, R. A., Winn, M. D., Long, F. & Vagin, A. A. (2011). *Acta Cryst.* D**67**, 355–367. 10.1107/S0907444911001314 PMC3069751 21460454

[bb12] Noller, H. F. & Woese, C. R. (1981). *Science*, **212**, 403–411. 10.1126/science.6163215 6163215

[bb13] Qian, B., Raman, S., Das, R., Bradley, P., McCoy, A. J., Read, R. J. & Baker, D. (2007). *Nature (London)*, **450**, 259–264. 10.1038/nature06249 PMC2504711 17934447

[bb14] Robertson, M. P., Chi, Y.-I. & Scott, W. G. (2010). *Methods*, **52**, 168–172. 10.1016/j.ymeth.2010.06.011 PMC2948636 20541014

[bb15] Robertson, M. P. & Scott, W. G. (2007). *Science*, **315**, 1549–1553. 10.1126/science.1136231 17363667

[bb16] Robertson, M. P. & Scott, W. G. (2008). *Acta Cryst.* D**64**, 738–744. 10.1107/S0907444908011578 PMC2507861 18566509

[bb17] Robertus, J. D., Ladner, J. E., Finch, J. T., Rhodes, D., Brown, R. S., Clark, B. F. C. & Klug, A. (1974). *Nature (London)*, **250**, 738–744. 10.1038/250546a0 4602655

[bb18] Serganov, A., Keiper, S., Malinina, L., Tereshko, V., Skripkin, E., Höbartner, C., Polonskaia, A., Phan, A. T., Wombacher, R., Micura, R., Dauter, Z., Jäschke, A. & Patel, D. J. (2005). *Nature Struct. Mol. Biol.* **12**, 218–224. 10.1038/nsmb906 PMC4692364 15723077

[bb19] Suddath, F. L., Quigley, G. J., McPherson, A., Sneden, D., Kim, J. J., Kim, S.-H. & Rich, A. (1974). *Nature (London)*, **248**, 20–24. 10.1038/248020a0 4594440

[bb2] Winn, M. D. *et al.* (2011). *Acta Cryst.* D**67**, 235–242.

